# The ALP score: a novel composite index for prognostic risk stratification to chemo-immunotherapy in advanced non-small cell lung cancer

**DOI:** 10.3389/fimmu.2026.1889854

**Published:** 2026-09-03

**Authors:** Yize Wang, Xiaolong Zhou, Yan Huang, Ting Zhong, Huan Yan, Juan Liang, Lianxi Song

**Affiliations:** 1 Department of Medical Oncology, Yiyang Central Hospital, Yiyang, Hunan, China; 2 Department of Ultrasound in Medicine, Yiyang Central Hospital, Yiyang, Hunan, China; 3 Early Clinical Trial Center, Hunan Cancer Hospital/The Affiliated Cancer Hospital of Xiangya School of Medicine, Central South University, Changsha, Hunan, China

**Keywords:** ALP score, immune checkpoint inhibitors, machine learning, non-small cell lung cancer, primary resistance

## Abstract

**Objective:**

This study aimed to investigate the value of a novel composite index, the ALP score (calculated as the product of lymphocyte count and serum albumin), in predicting the risk of early progression to chemotherapy combined with immune checkpoint inhibitors (ICIs) in patients with driver mutation-negative advanced non-small cell lung cancer (NSCLC).

**Methods:**

Clinical data from 433 patients with advanced NSCLC who received first-line chemoimmunotherapy were retrospectively collected. Independent risk factors for early progression (defined as progressive disease, PD) were identified using univariate and multivariate logistic regression analyses. The predictive power of the ALP score for PD was evaluated, and its nonlinear relationship with risk was explored using restricted cubic splines (RCS). Ten machine learning models and the SHAP interpretability technique were further employed to validate the importance of the ALP score.

**Results:**

Multivariate analysis identified the absence of liver metastasis and the absence of bone metastasis as independent protective factors against early progression, whereas lower lymphocyte count, lower albumin level, and lower Body Mass Index were identified as independent risk factors. The ALP score predicted PD with an AUC of 0.643, outperforming its individual components. Critically, multivariable analyses confirmed the ALP score as an independent predictor for both endpoints: patients in the highest score tertile had significantly lower risks of early progression (OR = 0.33, P = 0.001) and disease progression or death (HR = 0.65, P = 0.002) compared to the lowest tertile. RCS analysis revealed a significant nonlinear inverse relationship between the ALP score and PD risk (P<0.05). Machine learning models (best model AUC = 0.655) and SHAP analysis consistently confirmed the ALP score as a key predictive feature for disease progression.

**Conclusion:**

The ALP score, a composite index derived from routine laboratory parameters, serves as an accessible and practical prognostic marker associated with early progression risk and progression-free survival in patients with advanced NSCLC. Reflecting baseline systemic immune and nutritional readiness, it offers a practical and low-cost tool for clinical risk stratification in patients receiving chemo-immunotherapy.

## 1 Introduction

Lung cancer remains one of the most common malignancies with the highest incidence and mortality rates worldwide (1, 2), of which non-small cell lung cancer (NSCLC) accounts for more than 85% of all pathological types (3). Notably, the majority of patients are already diagnosed at an advanced stage with a very poor prognosis (1). For driver gene-negative advanced NSCLC, treatment has been replaced by immune checkpoint inhibitors (ICIs) combined with chemotherapy as the first-line standard, moving beyond traditional chemotherapy (4). Phase III clinical trials such as KEYNOTE-189 and IMpower150 have demonstrated that ICIs combined with chemotherapy significantly improve overall survival and progression-free survival (PFS) in patients (5–7).

However, primary resistance remains a serious challenge in clinical practice, with approximately 7%–27% of patients showing disease progression shortly after initial treatment with immunotherapy combinations (8, 9). The mechanisms are complex, involving factors at the tumor cell level (e.g., defects in antigen presentation, abnormalities in the interferon-γ signaling pathway), the tumor microenvironment (e.g., infiltration of immunosuppressive cells), and the host’s systemic status (e.g., systemic inflammation) (10–14). Currently, effective biomarkers for predicting primary resistance are still lacking. Although programmed death factor ligand 1 (PD-L1) expression and tumor mutational burden (TMB) are used clinically, they have limitations such as spatiotemporal heterogeneity and lack of standardized assays, and some patients with high PD-L1 expression still develop resistance (11). Because these static, tissue-based biomarkers cannot capture the dynamic nature of the tumor immune microenvironment (12), clinicians need more accessible tools to routinely evaluate a patient’s baseline immune and nutritional readiness for immunotherapy.

Recent studies have highlighted that the baseline immune and inflammatory status of the host is critical to the efficacy of ICIs. This conclusion has been supported by multiple lines of evidence: a lower peripheral lymphocyte count is associated with shorter PFS in cervical cancer patients, suggesting its potential as a prognostic marker (15); at the tumor site, a higher density of CD8^+^ lymphocytes correlates with a better response to PD-1 inhibitors in oral squamous cell carcinoma, underscoring the central role of lymphocytes in anti-tumor immunity (16). Meanwhile, indicators reflecting nutritional and systemic inflammation also hold predictive value. Both low serum albumin levels and a low Geriatric Nutritional Risk Index (GNRI) are associated with poorer outcomes following immunotherapy (17). Integrating lymphocyte count, a proxy for immune function, with serum albumin, a metabolic indicator, offers a more comprehensive baseline evaluation of a patient’s anti-tumor immune competence than either metric alone.

To address this clinical need, we developed the ALP score, a simple composite index calculated from baseline peripheral lymphocyte count and serum albumin levels. This score aims to comprehensively assess the immuno-nutritional status of patients and explore its value in prognostic risk stratification. To this end, we conducted a retrospective study designed to systematically analyze the association between baseline characteristics and the risk of early progression in patients with advanced NSCLC, and to further validate the efficacy of the ALP score in predicting this risk. Additionally, this study will employ machine learning models and the SHAP interpretability technique to further evaluate and elucidate the value of the ALP score in risk prediction.

## 2 Materials and methods

### 2.1 Enrolled patients

The data for this study were retrospectively collected from Yiyang Central Hospital and Hunan Cancer Hospital, covering the clinical information of 667 patients diagnosed with NSCLC without actionable driver mutations, who received immune checkpoint inhibitor treatment between January 1, 2020, and July 1, 2025. Patients included in this study had to meet the following criteria: first, histologically confirmed locally advanced or metastatic NSCLC; second, completion of routine blood and biochemical tests at baseline. Additionally, patients’ tumors had to harbor no targetable driver mutations, and they must have received first-line chemotherapy combined with immunotherapy upon diagnosis. Specifically, for patients with adenocarcinoma, driver-negative status was confirmed at baseline by the absence of actionable driver mutations (including EGFR, ALK, ROS1, RET, MET, NTRK, and BRAF V600E) evaluated via next-generation sequencing (NGS) panels or real-time PCR according to institutional clinical practice. Routine molecular testing was not required for patients with squamous cell carcinoma unless clinically indicated, in accordance with standard treatment guidelines. This study was approved by the Ethics Committee of Yiyang Central Hospital (approval No. 2026–JY-07).

**Figure 1:**
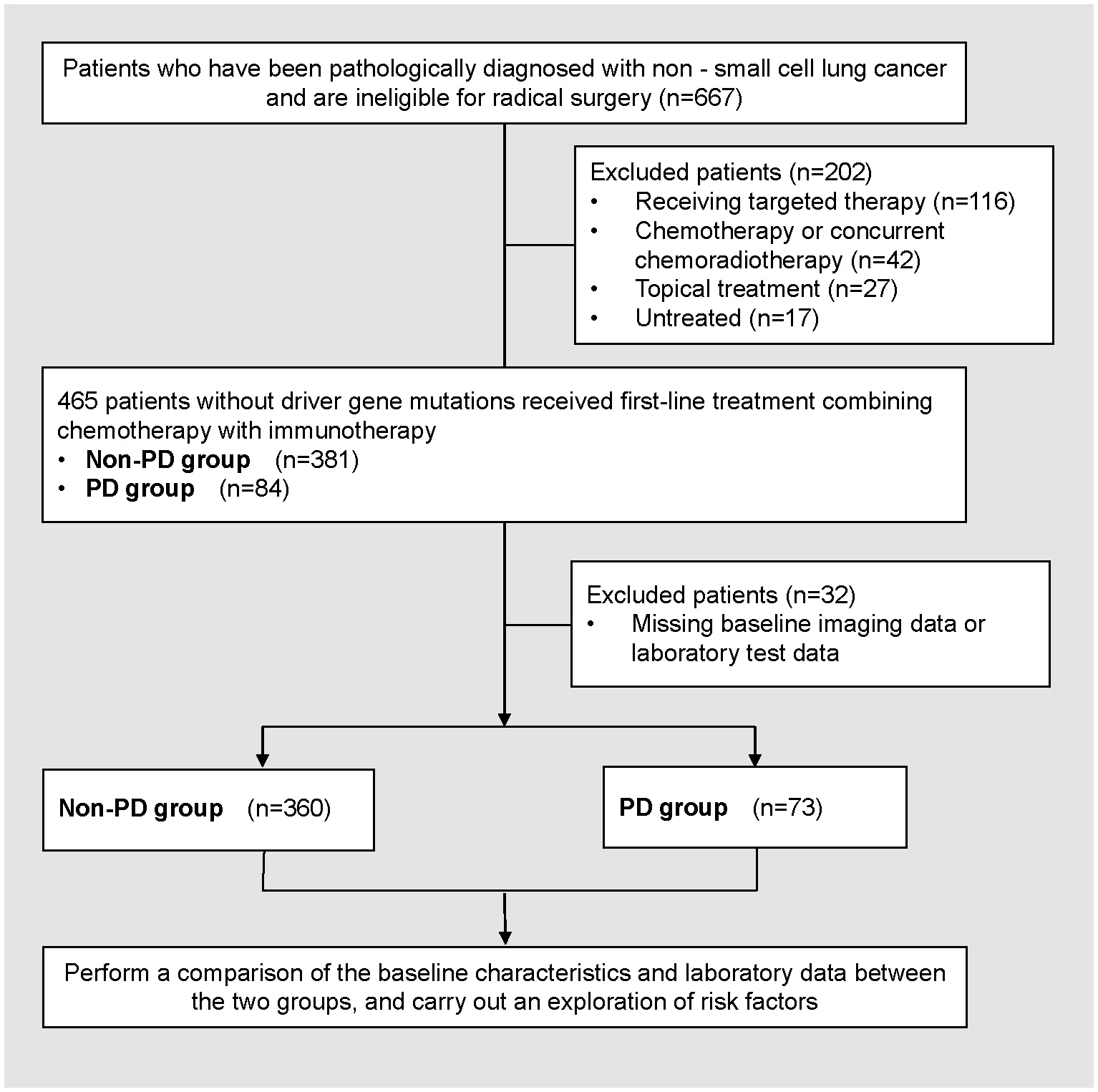
Study flowchart depicting patient selection from the initial database to the final analysis cohort. The initial database contained records of 465 patients who received ICIs. The flowchart starts with 667 patients after the application of broader inclusion criteria (pathologically diagnosed, ineligible for surgery), with subsequent exclusions leading to the final study population of 433 patients.

Patient screening and enrollment are detailed in **Figure 1**. Briefly, 667 patients with pathologically diagnosed NSCLC who were ineligible for radical surgery were considered. After excluding 202 patients who received targeted therapy (n=116), chemotherapy or concurrent chemoradiotherapy alone (n=42), topical treatment (n=27), or were untreated (n=17), 465 patients without driver gene mutations who received first-line chemoimmunotherapy were initially identified. However, 32 patients were further excluded due to missing baseline imaging or laboratory test data. Ultimately, 433 patients were included in the final analysis. These patients were categorized into the Non-Progressive Disease (Non-PD, n=360) group and the Progressive Disease (PD, n=73) group based on treatment response.

### 2.2 Assessment of chemotherapy and immunotherapy efficacy

The PD-1 inhibitors administered to patients were tislelizumab, camrelizumab, sintilimab, or pembrolizumab (at a dosage of 200 mg, intravenously, every 21 days). The chemotherapy regimens included pemetrexed combined with platinum for non-squamous NSCLC, and paclitaxel/nab-paclitaxel combined with platinum for squamous NSCLC. The selection of specific ICI agents and chemotherapy combinations was guided by institutional protocols, NCCN/CSCO guidelines and local health insurance reimbursement policies. Pharmacogenomic testing or drug-sensitivity assays were not routinely performed to guide regimen selection. Treatment response was strictly assessed according to the Response Evaluation Criteria in Solid Tumors (RECIST version 1.1). Baseline tumor assessment was performed before treatment initiation, followed by evaluations every 6 to 8 weeks until disease progression was confirmed. Specifically, efficacy was evaluated at baseline (Day 0) and thereafter every 2 treatment cycles (approximately 45–50 days) via computed tomography (CT) or magnetic resonance imaging (MRI). All imaging results were independently reviewed by two radiologists. Furthermore, each systematic response assessment was conducted independently by the attending physician. The key efficacy endpoints used in this study were defined as follows: the overall response rate (ORR) was defined as the proportion of patients who achieved a partial response (PR) or complete response (CR); the disease control rate (DCR) was defined as the proportion of patients with CR, PR, or stable disease (SD); and PFS was defined as the interval from the initiation of ICIs therapy to tumor progression, death from any cause, or the last known follow-up.

### 2.3 Acquisition of clinical characteristics and laboratory indicators

Data for all variables evaluated in this study were derived from patients’ inpatient electronic medical records. The collected data encompassed basic patient information (including age, sex, and body mass index), clinicopathological characteristics (such as TNM staging according to the American Joint Committee on Cancer 8th edition, metastatic sites, pathological type, molecular mutations, and PD-L1 expression level). Specifically, information on T stage and N stage was obtained for TNM staging. Laboratory parameters included platelet count, lymphocyte count, monocyte count, neutrophil count, eosinophil count, basophil count, total bilirubin, and human serum albumin level. Based on these, the ALP (calculated as the product of lymphocyte count and albumin level) was derived. All the aforementioned predictive indicators were objective data recorded in the EMR.

### 2.4 Model development and interpretation

Ten machine learning models were employed to predict the risk of progressive disease in non-small cell lung cancer patients, including Random Forest, Gradient Boosted Trees, Support Vector Machine, Logistic Regression, K-Nearest Neighbors, Partial Least Squares, Gradient Boosting Machine, Neural Network, Lasso Regression, and Adaptive Boosting. To interpret the machine learning predictions, we applied SHAP (SHapley Additive exPlanations) analysis. SHAP clarifies the model’s decision-making process by quantifying how much each baseline feature contributes to the predicted risk of disease progression, offering both cohort-level and patient-level insights. Furthermore, for each machine learning model, the Recursive Feature Elimination method was used to identify the optimal feature subset. The selected variables from RFE were visualized, and the importance score of each variable was presented in a bar chart.

### 2.5 Statistical analysis

All analyses were performed using R language (version 4.3.1). The machine learning models were developed and trained using the “caret” package (version 6.0.94), which provides a unified interface for various algorithms; models were built via the trainfunction with specified algorithm parameters, such as those for logistic regression, decision tree, random forest, support vector machine, K-nearest neighbors, XGBoost, gradient boosted trees, partial least squares, Lasso regression, and neural network. The discriminative performance of the models was evaluated using Receiver Operating Characteristic (ROC) curve analysis, and the Area Under the Curve (AUC) was reported. Continuous variables were presented as mean (standard deviation) or median (interquartile range) based on their distribution, and comparisons between groups were made using the independent samples t-test or the Wilcoxon rank-sum test. Categorical variables were summarized as frequencies and percentages, with comparisons made using the Chi-square test or Fisher’s exact test. Survival analysis was performed using the Kaplan-Meier method to estimate survival functions, with between-group differences assessed by the log-rank test; multivariable survival analysis was conducted using the Cox proportional hazards model, and the proportional hazards assumption was tested using Schoenfeld residuals. All tests were two-sided, and a P-value less than 0.05 was considered statistically significant.

## 3 Result

### 3.1 Clinicopathological characteristics and laboratory parameters associated with disease progression

A total of 433 patients with advanced NSCLC who received immune checkpoint inhibitors were enrolled in this study to identify factors associated with PD. As shown in **Table 1**, the baseline clinicopathological characteristics were well-balanced between the progressive disease (PD, n=73) and non-progressive disease (Non-PD, n=360) groups for most variables, including sex, age, and smoking history. However, univariate analysis indicated that patients in the PD group were more likely to have stage IV disease (93.15% vs. 79.44%, P = 0.006) and a significantly higher prevalence of bone (49.32% vs. 29.72%, P = 0.001), liver (23.29% vs. 8.89%, P<0.001), and brain metastases (17.81% vs. 9.44%, P = 0.036). Meanwhile, the comparison of laboratory indicators (**Table 2**) revealed that the PD group had significantly lower median levels of lymphocyte count (1.20 vs. 1.46 × 10^9^/L, P<0.001), eosinophil count (0.09 vs. 0.14 × 10^9^/L, P = 0.010), basophil count (0.03 vs. 0.04 × 10^9^/L, P = 0.012), albumin (41.20 vs. 42.40 g/L, P = 0.003), and blood urea nitrogen (4.02 vs. 4.83 mmol/L, P = 0.002) compared to the Non-PD group. These findings preliminarily suggest that advanced tumor burden, specific metastatic sites, and baseline laboratory parameters reflecting nutritional and immune status may be associated with the risk of disease progression.

**Table 1:** Comparison of clinicopathological characteristics between PD patients and non-PD patients (n, %).

Variables	Total (n = 433)	PD (n = 73)	Non-PD (n = 360)	*P*
Sex				0.476
Female	82 (18.94)	16 (21.92)	66 (18.33)	
Male	351 (81.06)	57 (78.08)	294 (81.67)	
Age				0.314
<59	214 (49.42)	40 (54.79)	174 (48.33)	
≥59	219 (50.58)	33 (45.21)	186 (51.67)	
Smoking				0.250
Former	331 (76.44)	52 (71.23)	279 (77.50)	
Never	102 (23.56)	21 (28.77)	81 (22.50)	
PS score				0.765
High(2)	18 (4.16)	4 (5.48)	14 (3.89)	
Low(0-1)	415 (95.84)	69 (94.52)	346 (96.11)	
Stage				0.006**
IIIB/IIIC	79 (18.24)	5 (6.85)	74 (20.56)	
IV	354 (81.76)	68 (93.15)	286 (79.44)	
N Stage				0.187
N0-N2	284 (65.59)	43 (58.90)	241 (66.94)	
N3	149 (34.41)	30 (41.10)	119 (33.06)	
Pleural or pericardial metastasis				0.203
With	69 (15.94)	8 (10.96)	61 (16.94)	
Without	364 (84.06)	65 (89.04)	299 (83.06)	
Bone metastasis				0.001**
With	143 (33.03)	36 (49.32)	107 (29.72)	
Without	290 (66.97)	37 (50.68)	253 (70.28)	
Liver metastasis				<0.001**
With	49 (11.32)	17 (23.29)	32 (8.89)	
Without	384 (88.68)	56 (76.71)	328 (91.11)	
Brain metastases				0.036*
With	47 (10.85)	13 (17.81)	34 (9.44)	
Without	386 (89.15)	60 (82.19)	326 (90.56)	
Histology				0.958
Adenocarcinoma	240 (55.43)	40 (54.79)	200 (55.56)	
Large cell carcinoma	6 (1.39)	1 (1.37)	5 (1.39)	
Squamous	187 (43.19)	32 (43.84)	155 (43.06)	
KRAS mutation				0.755
Unknown/Without	367 (84.76)	61 (83.56)	306 (85.00)	
With	66 (15.24)	12 (16.44)	54 (15.00)	
PD-L1 level				0.968
<1%	66 (15.24)	12 (16.44)	54 (15.00)	
≥50%	83 (19.17)	15 (20.55)	68 (18.89)	
1-49%	93 (21.48)	15 (20.55)	78 (21.67)	
Unknown	191 (44.11)	31 (42.47)	160 (44.44)	

PD, Progressive disease; Non-PD, Including complete response; partial response and stable disease.

*P <0.05; **P<0.01.

**Table 2:** Comparison of laboratory indicators between PD patients and non-PD patients [M (Q1, Q3)].

Variables	Total (n = 433)	PD (n = 73)	Non-PD (n = 360)	*P*
Neutrophil count (×10^9^/L)	4.58 (3.58, 6.12)	4.93 (3.68, 6.88)	4.56 (3.55, 5.96)	0.298
Lymphocyte count (×10^9^/L)	1.41 (1.14, 1.87)	1.20 (0.90, 1.69)	1.46 (1.20, 1.89)	<0.001**
Monocyte count (×10^9^/L)	0.58 (0.46, 0.77)	0.60 (0.45, 0.83)	0.58 (0.47, 0.76)	0.946
Eosinophil count (×10^9^/L)	0.13 (0.07, 0.25)	0.09 (0.04, 0.21)	0.14 (0.08, 0.25)	0.010*
Basophil count (×10^9^/L)	0.04 (0.02, 0.05)	0.03 (0.02, 0.04)	0.04 (0.02, 0.05)	0.012*
Platelet count (×10^9^/L)	260.00 (203.00, 319.00)	269.00 (214.00, 323.00)	254.50 (201.00, 319.00)	0.557
Total bilirubin (mg/dL)	10.45 (8.07, 14.04)	9.70 (7.80, 12.86)	10.57 (8.12, 14.18)	0.103
Albumin	42.10 (38.70, 44.60)	41.20 (36.90, 43.10)	42.40 (39.00, 44.80)	0.003**
Blood urea nitrogen	4.75 (3.74, 5.98)	4.02 (3.30, 5.51)	4.83 (3.88, 6.08)	0.002**
BMI (kg/m^2^)	28.12 (21.26, 34.40)	27.71 (19.22, 35.40)	28.38 (21.39, 34.33)	0.367

M, Median; Q1, 1st Quartile; Q3, 3st Quartile; PD, Progressive disease; Non-PD, Including complete response, partial response and stable disease.

*P <0.05; **P<0.01.

### 3.2 Independent risk factors in conventional statistical models for immune checkpoint inhibitor therapy in driver mutation-negative NSCLC patients

**Figure 2:**
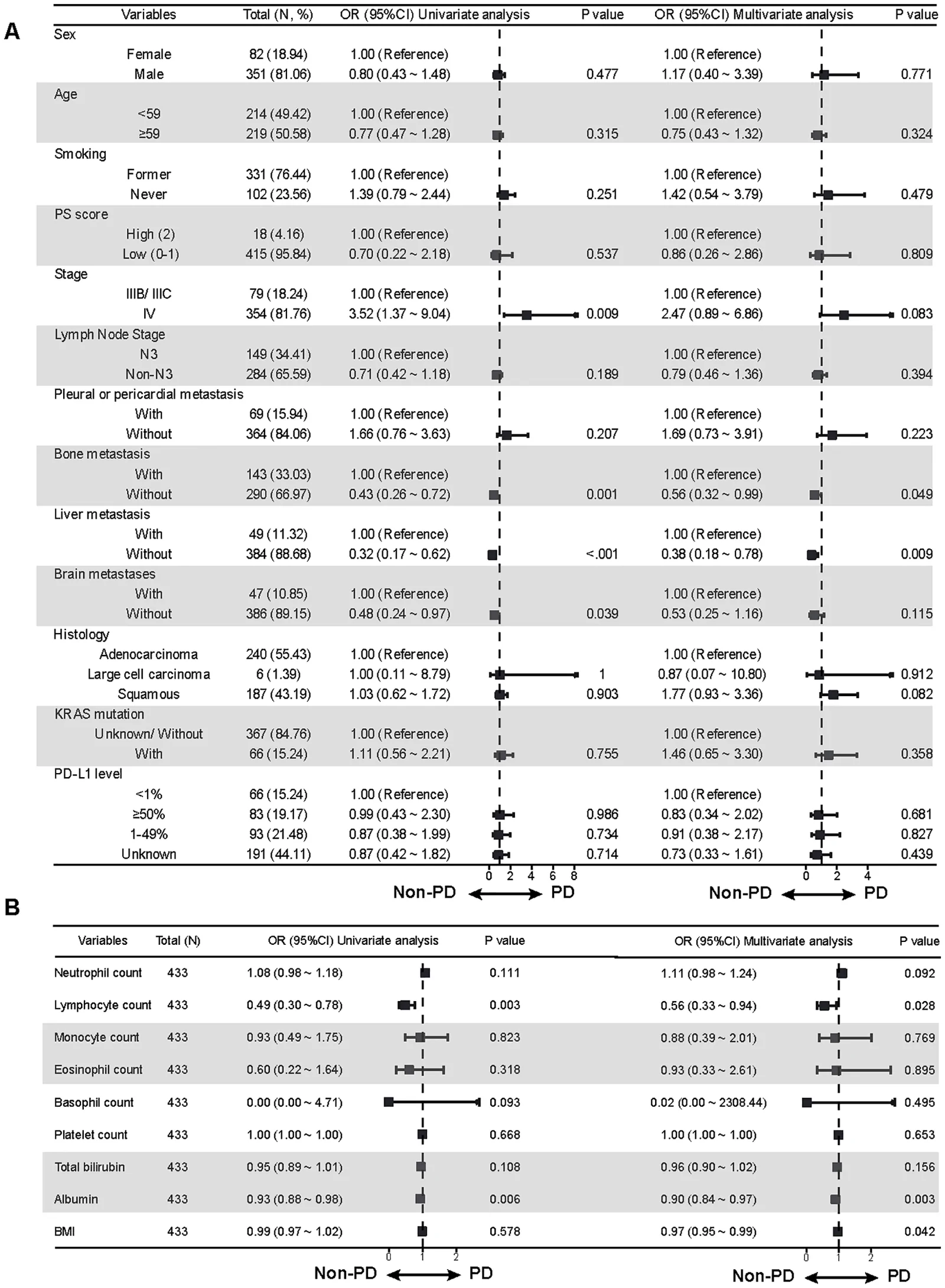
Multivariable logistic regression analysis of factors associated with early progression to chemoimmunotherapy. **(A, B)**. This image presents the results of a multivariable logistic regression analysis, demonstrating the independent risk factors associated with early progression (defined as progressive disease, PD) to chemoimmunotherapy in patients with advanced NSCLC.

Based on the entire cohort, we investigated the independent risk factors for early progression (risk of PD) to chemotherapy combined with immunotherapy in NSCLC. Univariate logistic regression analysis revealed that stage IV disease, bone metastasis, liver metastasis, brain metastasis, along with lower lymphocyte count, eosinophil count, basophil count, albumin, and blood urea nitrogen levels were significantly associated with PD. Subsequently, variables with a P-value < 1 from the univariate analysis were included in a multivariate logistic regression model. The results (**Figure 2**) ultimately identified the absence of liver metastasis (OR = 0.38, 95% CI: 0.18-0.78, P = 0.009) and the absence of bone metastasis (OR = 0.56, 95% CI: 0.32-0.99, P = 0.046) as independent protective factors against early progression. Conversely, lower baseline lymphocyte count (OR = 0.56, 95% CI: 0.33-0.94, P = 0.028), albumin level (OR = 0.90, 95% CI: 0.84-0.97, P = 0.003), and body mass index (OR = 0.97, 95% CI: 0.95-0.99, P = 0.042) were independently associated with an increased risk of early progression. It is noteworthy that after adjusting for other variables, stage IV disease showed a trend towards increased risk of PD, with marginal statistical significance (OR = 2.47, 95% CI: 0.89-6.86, P = 0.083).

**Figure 3:**
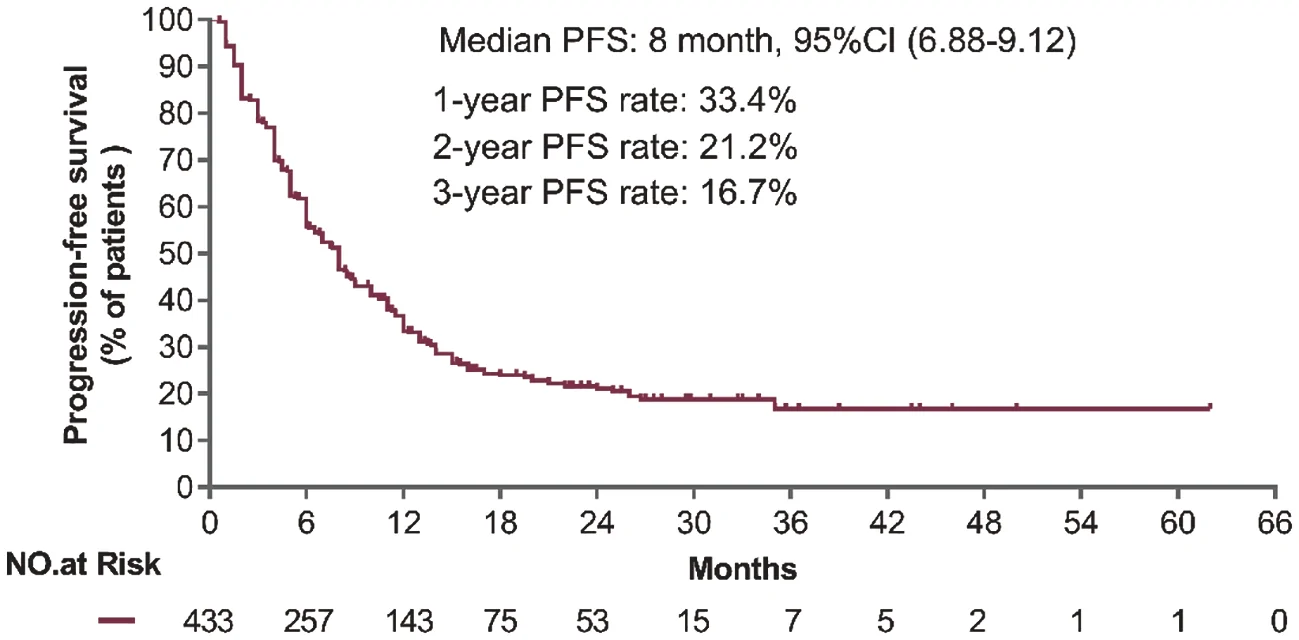
Progression-free survival of all patients treated with immune checkpoint inhibitors in driver mutation-negative NSCLC.

**Figure 4:**
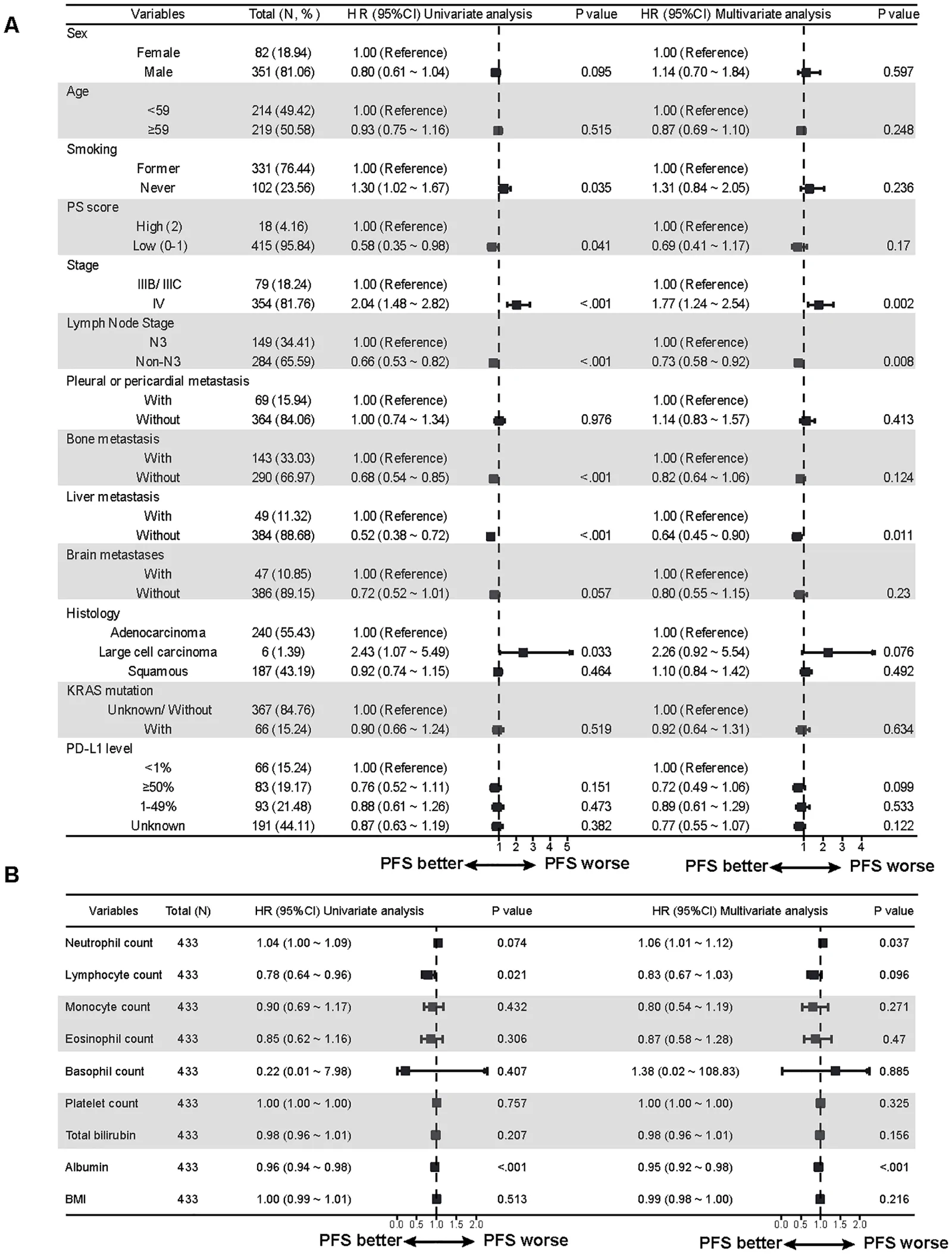
Univariate and multivariate Cox regression analyses of factors associated with PFS. **(A)** Forest plot showing the association between clinicopathological characteristics and PFS. **(B)** Forest plot showing the association between laboratory parameters and PFS. The analyses present HRs with 95% CIs from both univariate and multivariate Cox proportional hazards models.

**Figure 5:**
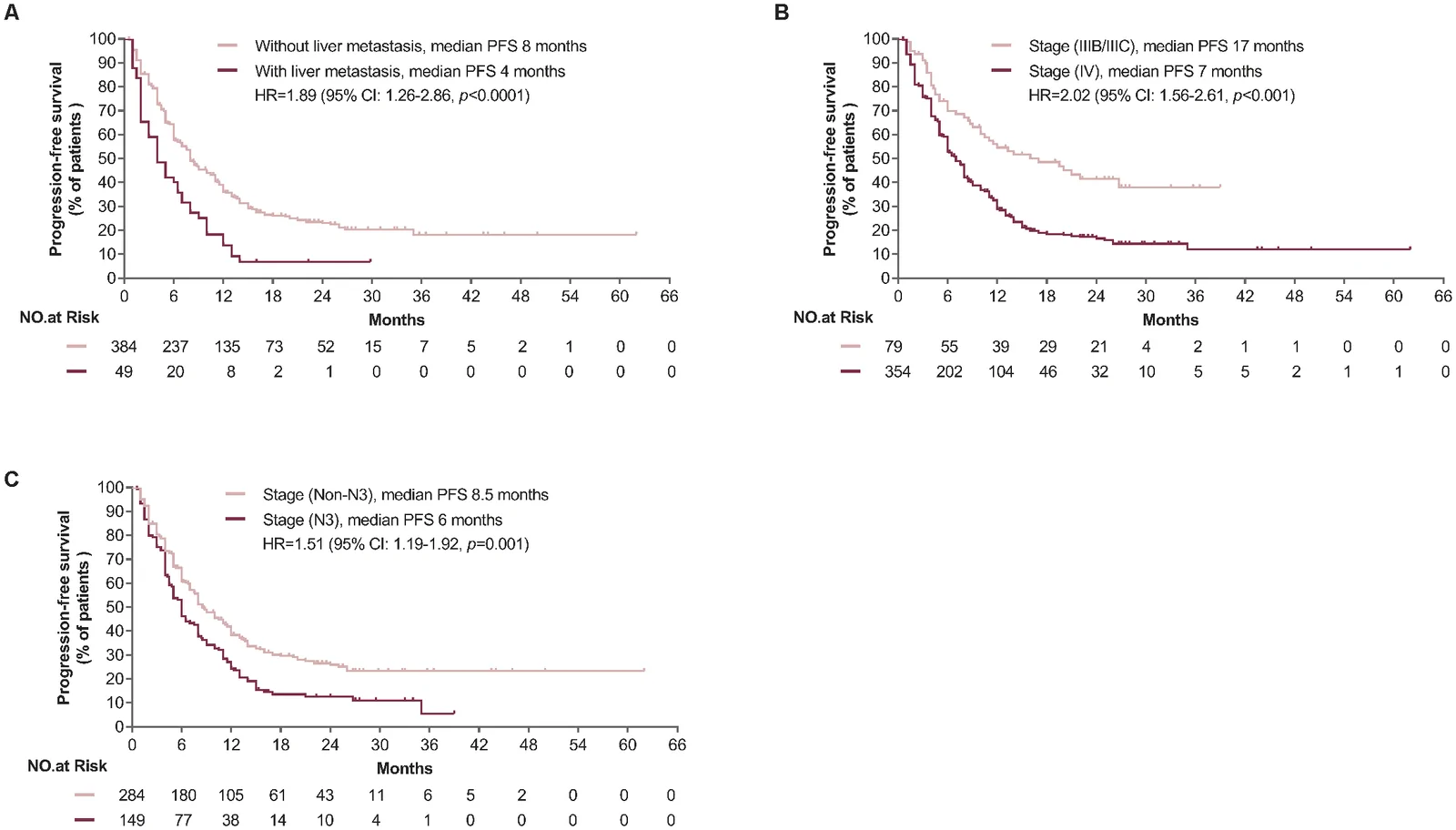
Forest plots of multivariate Cox regression analyses identifying independent prognostic factors for PFS. **(A–C)** Forest plots display the HRs and 95% CIs from the multivariate Cox proportional hazards regression analysis for **(A)** liver metastasis, **(B)** disease stage, **(C)** N stage associated with PFS.

Therefore, we performed a comprehensive survival outcome analysis for patients diagnosed with driver mutation-negative NSCLC who received ICIs therapy. As shown in the Kaplan-Meier curve in **Figure 3**, the median PFS for the entire cohort was 8.0 months (95% confidence interval: 6.88 - 9.11), with 1-year, 2-year, and 3-year PFS rates of 33.4%, 21.2%, and 16.7%, respectively. To identify independent prognostic factors, we conducted Cox proportional hazards regression analyses. As **Figure 4** shown, univariate analysis revealed that a low performance status (PS) score (0-1), non-N3 lymph node metastasis, absence of bone metastasis, and absence of liver metastasis were associated with better PFS, while stage IV disease, never smoking, and large cell carcinoma histology indicated a poorer prognosis. These factors were included in a multivariate analysis, which ultimately identified liver metastasis and disease stage as independent factors influencing PFS; the results are presented in forest plots (**Figures 5A–C**).

### 3.3 Association between the ALP score and risk of disease progression

**Figure 6:**
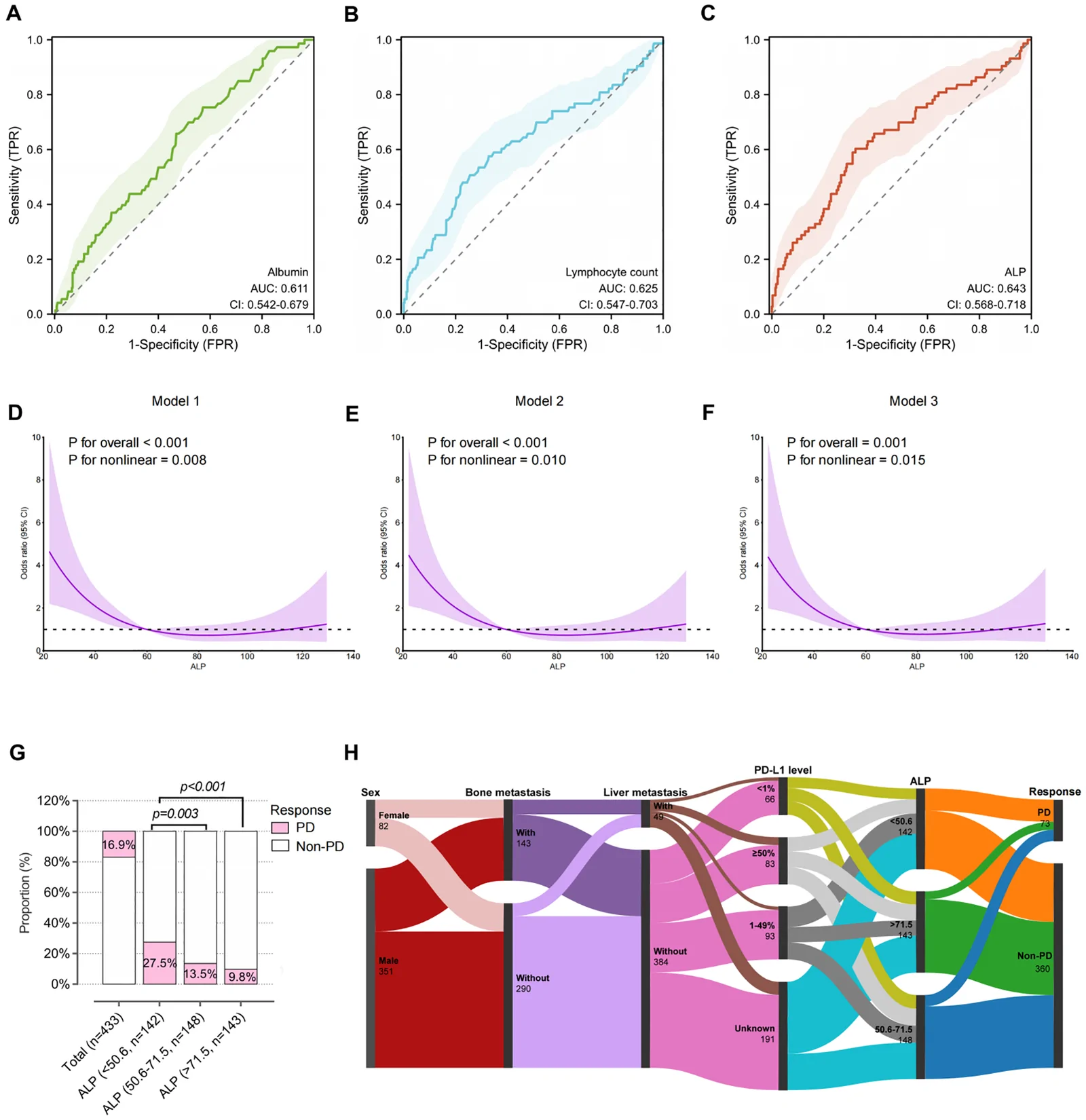
Predictive performance and clinical utility of the ALP score for early progression (progressive disease, PD). **(A–C)** ROC curves of and albumin level **(A)**, lymphocyte count **(B)** and APL score **(C)** alone for predicting PD. **(D–F)** Restricted cubic spline analyses showing the non-linear relationship between the ALP score and the risk of PD in the crude **(D)**, minimally adjusted **(E)**, and fully adjusted **(F)** models. **(G)** Bar graph illustrating the proportion of patients with PD across tertiles of the ALP score. **(H)** Sankey diagram visualizing the flow and relationships between patient characteristics (sex, bone metastasis, liver metastasis, PD-L1 level), ALP score strata, and disease progression outcomes.

Building upon the identification of lymphocyte count and albumin as independent risk factors from the multivariate logistic regression analysis, we further evaluated their predictive power for PD as continuous variables. The AUCs for lymphocyte count and albumin in predicting PD were 0.625 and 0.611, respectively (**Figures 6A, B**). To comprehensively assess these two parameters, we constructed the ALP score, defined as the product of lymphocyte count and albumin. The predictive AUC of the ALP score for PD increased to 0.643 (95% CI: 0.568-0.718) (**Figure 6C**). We then employed RCS analysis to explore the dose-response relationship between the ALP score and PD risk. A significant non-linear relationship was observed (P for overall <0.05, P for non-linear <0.05) (**Figure 6D**). This significant non-linear association persisted after adjusting for sex, age, and smoking status (Model 2, **Figure 6E**), and further after additional adjustment for PS score, stage, histology, and PD-L1 level (Model 3, **Figure 6F**) (P<0.05). To quantify the risk, patients were stratified into tertiles based on the ALP score. The proportion of PD was significantly higher in the lowest ALP tertile (<50.6) compared to the middle tertile (50.6-71.5) (27.5% vs. 13.5%, p=0.003) and the highest tertile (>71.5) (27.5% vs. 9.8%, p<0.001) (**Figure 6G**). Finally, a Sankey diagram was used to visualize the complex interrelationships between patient sex, bone metastasis status, liver metastasis status, PD-L1 level, ALP strata, and disease progression outcomes (**Figure 6H**).

Multivariable logistic regression analysis confirmed that a higher baseline ALP score was an independent protective factor against early progression (**Table 3**). When compared to the lowest ALP tertile (Q1, <50.6), patients in the Q2 (50.6-71.5) and Q3 (>71.5) tertiles had significantly reduced risk of early progression in the fully adjusted model (Model 3), with odds ratios (ORs) of 0.47 (95% CI: 0.25-0.87, P = 0.015) and 0.33 (95% CI: 0.16-0.64, P = 0.001), respectively. A significant dose-response relationship was observed (P for trend <0.05), indicating that the ALP score is a significant negative predictor of early progression to immunotherapy.

**Table 3:** Multivariable logistic regression analyses of the association between ALP score tertiles and the risk of early progression (progressive disease).

Variables	Model 1		Model 2		Model 3	
	OR (95% CI)	*P*	OR (95% CI)	*P*	OR (95% CI)	*P*
ALP						
Q1 (<50.6)	1.00 (Reference)		1.00 (Reference)		1.00 (Reference)	
Q2 (50.6-71.5)	0.42 (0.23 ~ 0.76)	0.004	0.43 (0.23 ~ 0.78)	0.006	0.47 (0.25 ~ 0.87)	0.015
Q3 (>71.5)	0.29 (0.15 ~ 0.56)	<.001	0.30 (0.15 ~ 0.58)	<.001	0.33 (0.16 ~ 0.64)	0.001

OR, Odds Ratio; CI, Confidence Interval.

Model 1: Crude.

Model 2: Adjust: Sex, Age, Smoking.

Model 3: Adjust: Sex, Age, Smoking, PS score, Stage, Histology, PD-L1 level.

The aforementioned findings indicate that the ALP score, as a new and comprehensive metric, holds modest but statistically significant predictive value in identifying the risk of early progression in non-small cell lung cancer patients treated with chemotherapy combined with immunotherapy, potentially offering a practical tool for clinical risk stratification.

### 3.4 Assessment of ALP’s predictive value using machine learning and SHAP interpretation

**Figure 7:**
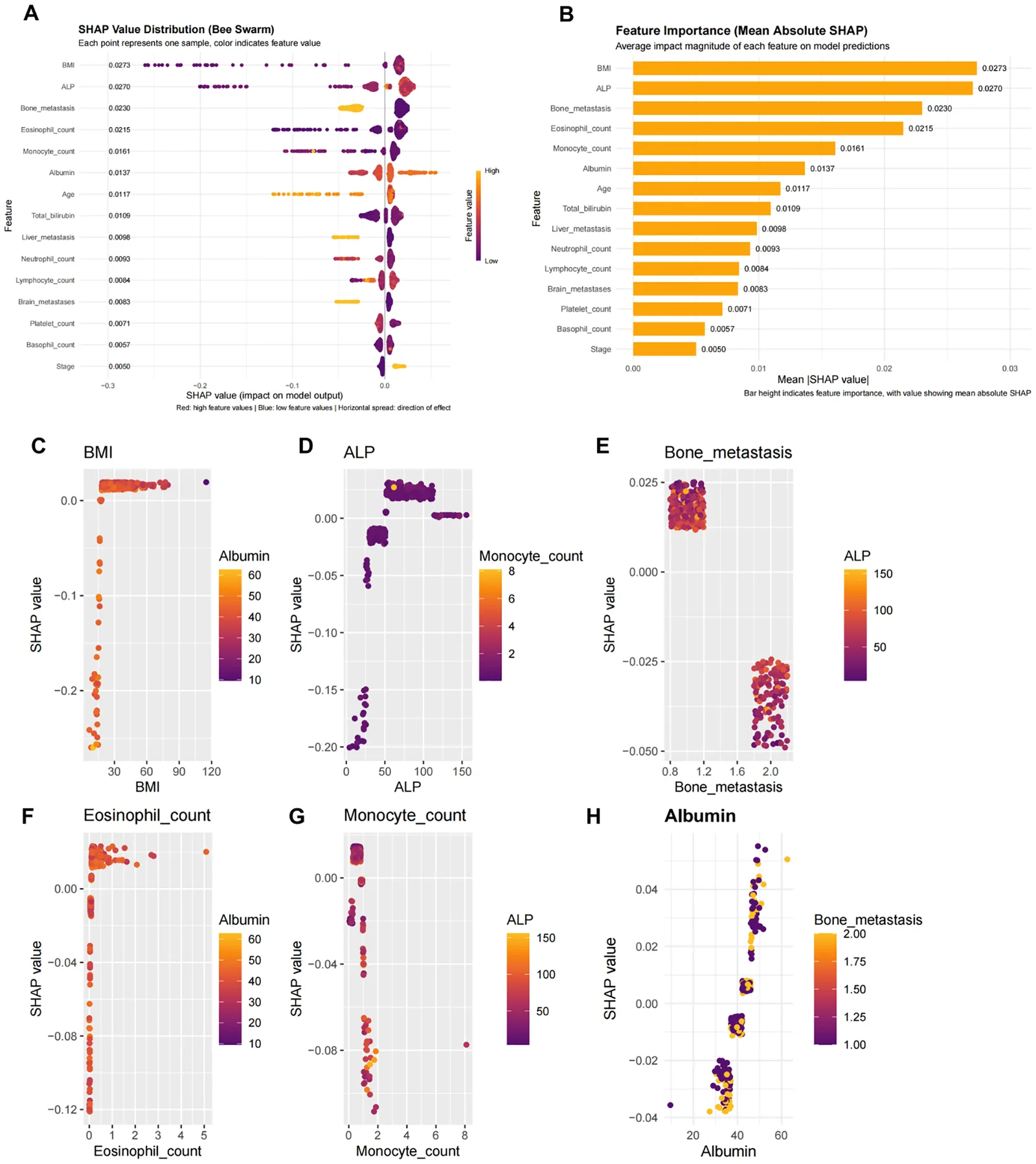
Interpretation of the optimal machine learning model using SHAP to assess the predictive value of key features for disease progression. **(A)** SHAP summary plot showing the distribution and impact of the top features on the model’s predictions. Each point represents a patient, and the color indicates the feature value (e.g., high or low ALP score). The plot reveals that low BMI, low ALP score, and the presence of bone metastasis are associated with an increased risk of disease progression (higher SHAP value). **(B)** SHAP bar plot ranking the mean absolute SHAP value of the top predictive features, indicating their overall importance in the model. The order of importance is BMI, ALP score, bone metastasis, eosinophil count, monocyte count, and albumin level. **(C–H)** SHAP dependence plots illustrating the nonlinear relationship between the SHAP value (impact on prediction) and the actual value of key features: **(C)** BMI, **(D)** ALP score, **(E)** bone metastasis, **(F)** eosinophil count, **(G)** monocyte count, and **(H)** albumin level. For example, the ALP score exhibits a positive correlation with the outcome, reaching its peak predictive contribution near a value of 50. Collectively, these interpretability analyses demonstrate that the ALP score is a key predictor in the model and validate its consistent and significant association with the risk of disease progression.

To further evaluate the predictive value of ALP, we constructed a risk prediction model for disease progression (with PD as the positive event) using ten machine learning methods. Among them, the Boosting model performed the best (AUC: 0.655; **Supplementary Figure 2A**). To ensure the clinical transparency of our machine learning predictions, we used SHAP analysis to delineate how individual baseline features—particularly the ALP score—drove the model’s output. In the global interpretation, the SHAP summary plot (**Figure 7A**) revealed that low BMI, low ALP score, and the presence of bone metastasis were significantly associated with an increased risk of disease progression. The SHAP bar plot (**Figure 7B**) further ranked the key predictive variables in descending order of importance: BMI, ALP score, bone metastasis, eosinophil count, monocyte count, and albumin level. Additionally, SHAP dependence plots (**Figures 7C–H**) uncovered nonlinear effects of key features: BMI showed a positive correlation with albumin and reached peak predictive contribution at around 22; ALP also exhibited a positive correlation with monocyte count and achieved maximum contribution near 50. For local interpretation, the SHAP waterfall plot for the 21st non-PD patient (**Supplementary Figure 2B**) illustrated the direction and magnitude of each feature’s contribution to the prediction. For instance, ALP = 111 contributed a positive influence of +0.0285, while BMI = 12.1 contributed a negative influence of –0.188, clearly demonstrating the model’s decision logic at the individual level. These findings suggest that ALP is an important factor in predicting the risk of disease progression, demonstrating consistent predictive contributions in both global and local interpretations. Together with the model interpretability analysis, these results further support the potential clinical utility of ALP in risk assessment.

### 3.5 Survival analysis and prognostic factors

**Figure 8:**
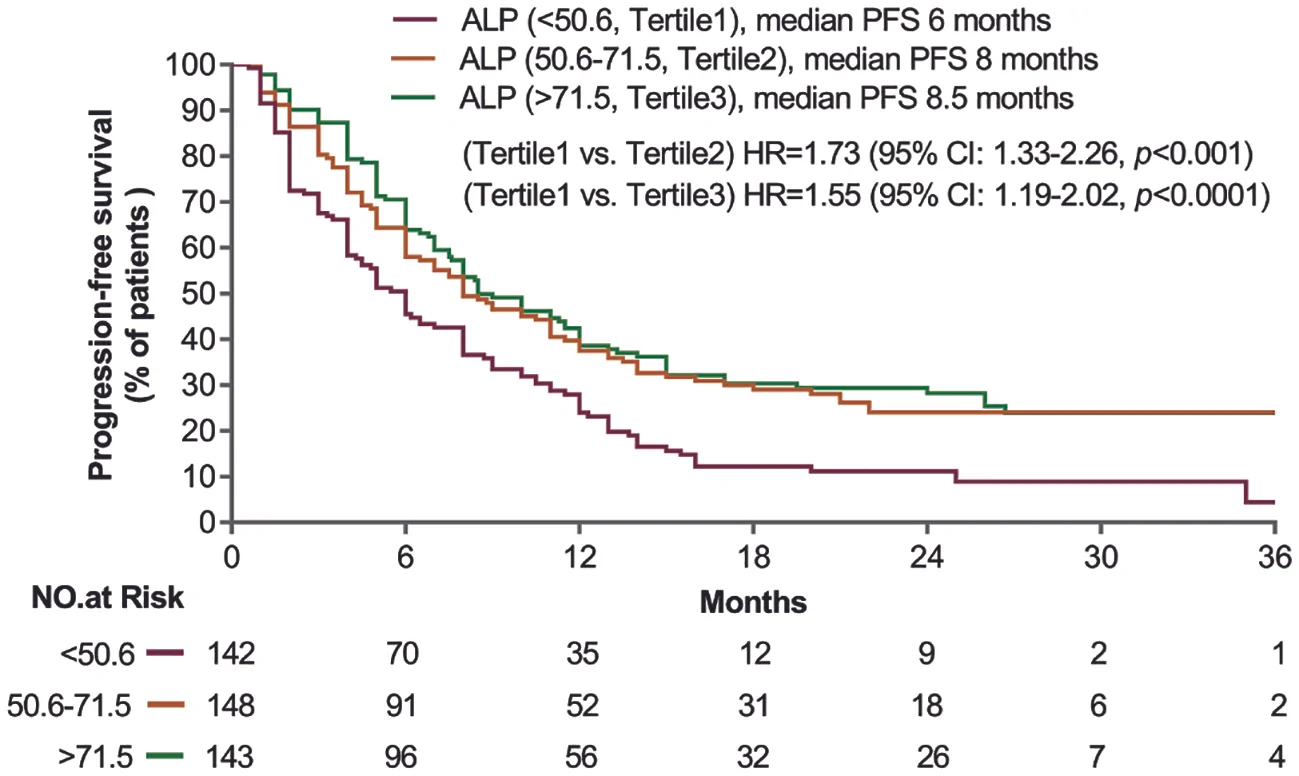
Prognostic value of the ALP score for PFS. Kaplan-Meier curves compare PFS between patients stratified by tertiles of the ALP score. Patients in the higher ALP score tertiles had significantly superior PFS compared to those in the lowest tertile (Log-rank P < 0.001).

We further explored the prognostic value of the ALP score (the product of lymphocyte count and albumin). As shown in **Figure 8**, when patients were stratified by the median ALP score, those in the high ALP score group had significantly superior PFS compared to the low score group (Log-rank P < 0.001). This score also demonstrated good discriminative ability in predicting 1-year, 2-year, and 3-year PFS (**Figure 8**). Multivariable Cox regression analysis confirmed that a higher baseline ALP score was an independent predictor for superior PFS (**Table 4**). Compared to patients in the lowest ALP score tertile (Q1, <50.6), those in the middle (Q2, 50.6-71.5) and highest (Q3, >71.5) tertiles exhibited significantly reduced risks of disease progression or death, with hazard ratios (HRs) of 0.73 (95% CI: 0.56-0.95, P = 0.019) and 0.65 (95% CI: 0.50-0.86, P = 0.002) in the fully adjusted model, respectively. A significant dose-response relationship was observed (P for trend <0.05), underscoring the ALP score as an independent prognostic biomarker for PFS in patients receiving chemoimmunotherapy. These findings collectively suggest that the ALP score serves as a significant prognostic biomarker, and along with disease stage and liver metastasis, provides valuable stratification for PFS in NSCLC patients treated with ICIs.

**Table 4:** Multivariable Cox regression analyses of the association between ALP score tertiles and PFS.

Variables	Model 1		Model 2		Model 3	
	HR (95%CI)	*P*	HR (95%CI)	*P*	HR (95%CI)	*P*
ALP						
Q1 (<50.6)	1.00 (Reference)		1.00 (Reference)		1.00 (Reference)	
Q2 (50.6-71.5)	0.66 (0.51 ~ 0.85)	0.002	0.66 (0.51 ~ 0.86)	0.002	0.73 (0.56 ~ 0.95)	0.019
Q3 (>71.5)	0.59 (0.45 ~ 0.77)	<.001	0.60 (0.46 ~ 0.78)	<.001	0.65 (0.50 ~ 0.86)	0.002

HR, Hazard Ratio; CI, Confidence Interval.

Model 1: Crude.

Model 2: Adjust: Sex, Age, Smoking.

Model 3: Adjust: Sex, Age, Smoking, PS score, Stage, Histology, PD-L1 level.

## 4 Discussion

This study integrated baseline clinical characteristics with routine laboratory indicators to explore early progression risk factors for chemotherapy combined with immunotherapy in patients with driver gene-negative advanced NSCLC. Our key finding is that the ALP score—a composite index derived from the product of lymphocyte count and serum albumin—serves as an independent predictor significantly associated with the risk of early progression. Both multivariate analysis and interpretable machine learning models consistently validated the importance of the ALP score and revealed a non-linear relationship with risk, offering a potentially accessible and low-cost tool for identifying high-risk populations.

An important strength of this study lies in the mutual validation achieved through multiple analytical methods. Traditional multivariate logistic regression, after adjusting for confounding factors, confirmed the independent predictive value of the ALP score. Furthermore, we employed ten machine learning models for prediction, among which the best-performing Boosting model (AUC = 0.655) reaffirmed through SHAP analysis—an interpretability technique—that the ALP score is one of the key features from a data-driven perspective. SHAP analysis not only globally identified a low ALP score as a major driver of high-risk predictions (**Figures 4A, B**) but also, through its dependence plots (**Figures 4C–H**), uncovered a non-linear relationship between the ALP score and risk, indicating a sharp increase in risk when the ALP score falls below a specific threshold (approximately 50). This finding transcends the assumptions of traditional linear models, offering a more refined depiction of risk dynamics and providing a potential basis for setting clinical intervention thresholds.

In terms of clinical translation, the ALP score offers practical value as an accessible and low-cost prognostic tool. Although its discriminative performance is modest (AUC = 0.643), the ALP score relies entirely on two routinely monitored laboratory parameters, making it inexpensive, simple to calculate, and easily implementable across diverse healthcare settings. Rather than replacing established tissue-based biomarkers such as PD-L1 expression, the ALP score complements them by providing a holistic snapshot of the patient’s baseline immuno-nutritional and physiological status. This simple index serves as a valuable clinical prognostic aid, particularly in resource-limited settings or scenarios where tissue samples are insufficient and PD-L1 testing is delayed or unavailable (18, 19). Second, after stratifying patients by tertiles, we observed a significantly higher proportion of disease progression in those with an ALP score < 50.6 compared to those with a score > 71.5 (27.5% vs. 9.8%), indicating that the ALP score can effectively identify high-risk individuals for early progression before treatment initiation. For these patients, clinicians might consider more proactive interventions, such as enhanced nutritional support to improve baseline status, exploring combination therapies with different mechanisms of action (e.g., anti-angiogenic agents), or implementing more frequent imaging surveillance for early progression detection.

The predictive power of the ALP score is biologically plausible. Peripheral lymphocytes serve as the primary source of anti-tumor effector T cells, making their baseline count a direct reflection of systemic immune competence. Low lymphocyte levels often indicate immune dysfunction, such as exhaustion or senescence, which can impair the response to ICIs (21). Specifically, in NSCLC patients, higher lymphocyte counts before and during treatment are associated with improved PFS and overall survival (OS), underscoring lymphocyte abundance as a key indicator of immune competence (20). A large real-world study further confirmed that a higher baseline absolute lymphocyte count correlates with better OS, while a low count suggests poorer prognosis (21). Additionally, the degree of lymphocyte infiltration within the tumor microenvironment (TME) is also linked to treatment efficacy. In NSCLC, high intratumoral density of T cells and B cells is associated with durable clinical benefit, particularly highlighting the importance of lymphocyte infiltration in patients with low PD-L1 expression (22). These findings illustrate that lymphocyte quantity and functional status, both peripherally and within the TME, collectively constitute the foundation of anti-tumor immunity. Future studies combining peripheral immuno-nutritional profiling with direct intratumoral immune analysis are needed to confirm whether baseline peripheral lymphocyte metrics truly reflect functional anti-tumor immunity within the local microenvironment.

Serum albumin level is a key indicator of systemic nutritional status and inflammatory levels. Hypoalbuminemia is often associated with a chronic inflammatory state, while adequate nutrition is crucial for the effective proliferation and function of immune cells. A large real-world study clearly showed that higher baseline serum albumin concentration is significantly associated with better OS in patients with advanced solid tumors receiving ICIs, establishing its role as a positive prognostic marker (21). This association is further elucidated in NSCLC patients. Research found that in patients receiving ICI monotherapy, pretreatment albumin levels and an early decrease in albumin (≥10% within a median of 21 days) were independently associated with OS; patients with early albumin reduction had significantly shorter OS (23). Notably, this association was not observed in patients receiving chemoimmunotherapy, suggesting that dynamic changes in albumin might be a specific predictor for ICI monotherapy efficacy (23). Furthermore, the integration of albumin into more complex prognostic scores underscores its value. The Advanced Lung cancer Inflammation Index (ALI), which includes albumin, body mass index, and the neutrophil-to-lymphocyte ratio, demonstrated that a high ALI score was significantly associated with longer OS, higher objective response rate, and longer treatment duration in ICI monotherapy, outperforming other established biomarkers (24). This indicates that albumin, as a core component, is key to assessing host inflammatory and nutritional status for predicting immunotherapy outcomes.

In this context, the ALP score serves as a dual surrogate for immunological readiness (lymphocyte count) and metabolic reserve (albumin). A low score typically points to concurrent immune exhaustion and systemic inflammation, which compromises the patient’s ability to mount an effective anti-tumor response and thereby increases the risk of early treatment failure. This biological rationale aligns with our observation that the predictive power of the ALP score (AUC = 0.643) surpassed that of its individual components.

This study has several limitations that warrant consideration. First, its retrospective observational design inherently carries risks of selection bias and unmeasured confounding; while multivariable regression and machine learning models were applied, contemporary causal inference approaches—such as propensity score matching (PSM) or inverse probability weighting (IPW)—could further minimize confounding in future studies. Second, internal cross-validation and SHAP interpretability in machine learning algorithms do not substitute for independent external cohort validation. Third, the ALP score reflects baseline systemic immuno-nutritional readiness rather than direct tissue-level tumor microenvironment or cellular resistance mechanisms, making mechanistic inferences preliminary. Fourth, the lack of PD-L1 expression data in approximately 44% of cases limited our ability to directly compare the ALP score against this key biomarker in multivariate models, though this also highlights the utility of the ALP score in scenarios with incomplete data. Fifth, molecular testing was not uniformly applied across histological subtypes, as routine testing was restricted to adenocarcinomas while performed selectively in squamous cell carcinomas per practice guidelines, potentially introducing minor residual selection variance. Finally, defining primary resistance strictly as progression at the first assessment may overlook rare dynamic responses like pseudoprogression, which are better captured by broader 6-month windows.

Future research should therefore focus on conducting large-scale, multi-center prospective clinical studies to further validate the predictive performance of the ALP score across broader populations. Concurrently, exploring the relationship between the ALP score and deeper biomarkers, such as tumor genomics and tumor microenvironment characteristics, will help to more comprehensively elucidate the integrated mechanisms of early progression.

In conclusion, this study demonstrates that the ALP score, constructed from routine laboratory indicators, is an accessible and practical tool for prognostic risk stratification to immunotherapy in patients with NSCLC. It reflects the host’s underlying immune and nutritional status and is corroborated by machine learning analysis. By effectively predicting early progression to chemoimmunotherapy, this easily accessible score offers a practical tool to help clinicians stratify risk and tailor early treatment decisions in advanced NSCLC.

## Data Availability

The raw data supporting the conclusions of this article will be made available by the authors, without undue reservation.
