# Supplementary material for: The ALP score: a novel composite index for prognostic risk stratification to chemo-immunotherapy in advanced non-small cell lung cancer
Source: Front Immunol. 2026 Sep 3;17:1889854. doi: 10.3389/fimmu.2026.1889854 (PMC13582344; doi:10.3389/fimmu.2026.1889854)
Supplement: Supplementary file 1 [file DataSheet1.pdf]

Figure S1

A

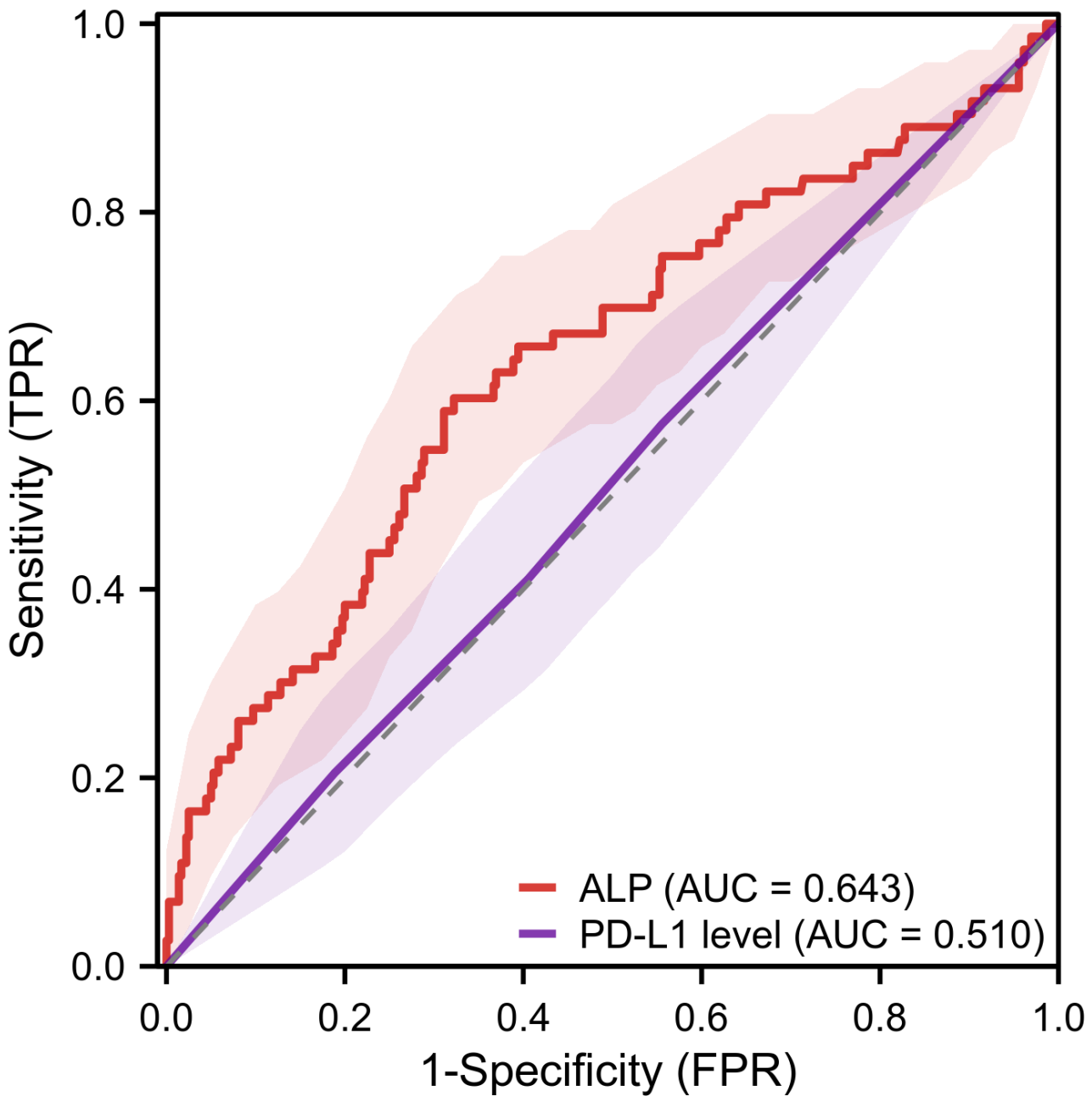

1.0
0.8
0.6
0.4
0.2
0.0
Sensitivity (TPR)
0.0
0.2
0.4
0.6
0.8
1.0
1-Specificity (FPR)
ALP (AUC = 0.643)
PD-L1 level (AUC = 0.510)
