# Supplementary material for: The ALP score: a novel composite index for prognostic risk stratification to chemo-immunotherapy in advanced non-small cell lung cancer
Source: Front Immunol. 2026 Sep 3;17:1889854. doi: 10.3389/fimmu.2026.1889854 (PMC13582344; doi:10.3389/fimmu.2026.1889854)
Supplement: Supplementary file 2 [file DataSheet2.pdf]

Figure S2

A

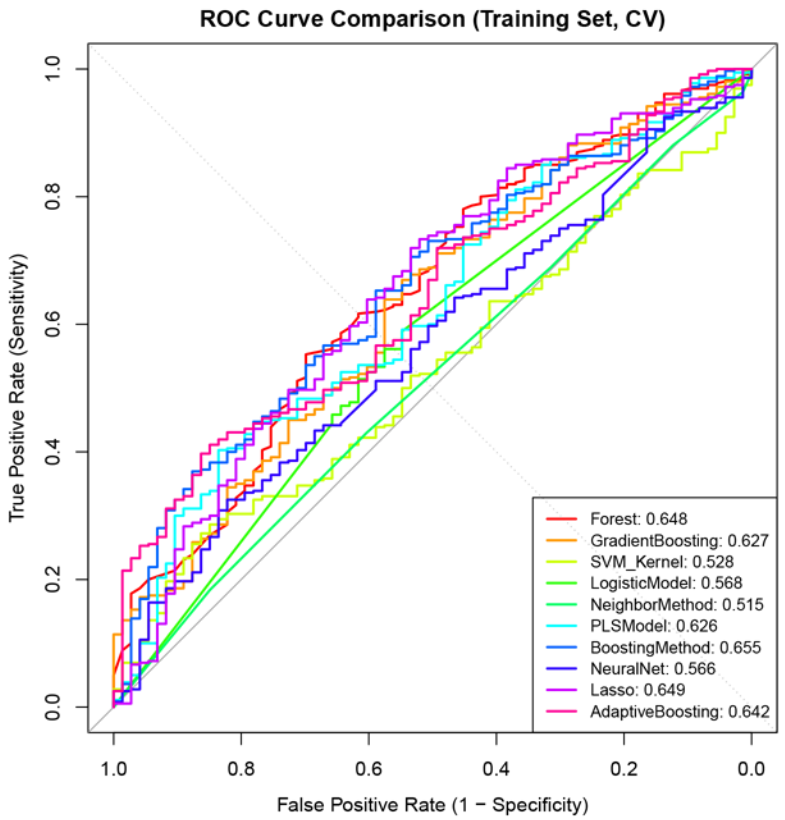

ROC Curve Comparison (Training Set, CV)
True Positive Rate (Sensitivity)
False Positive Rate (1 − Specificity)
0.0
0.2
0.4
0.6
0.8
1.0
Forest: 0.648
GradientBoosting: 0.627
SVM_Kernel: 0.528
LogisticModel: 0.568
NeighborMethod: 0.515
PLSModel: 0.626
BoostingMethod: 0.655
NeuralNet: 0.566
Lasso: 0.649
AdaptiveBoosting: 0.642

B

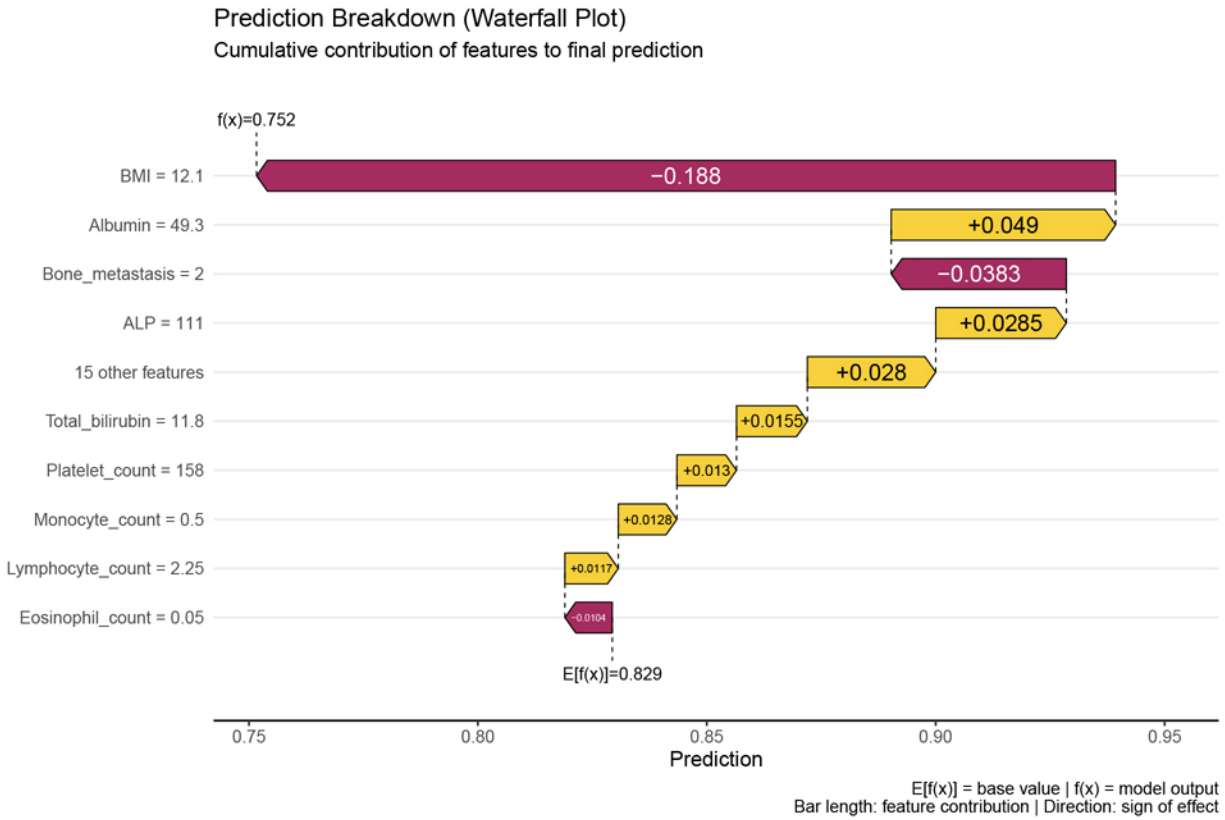

Prediction Breakdown (Waterfall Plot)
Cumulative contribution of features to final prediction
f(x)=0.752
BMI = 12.1
−0.188
Albumin = 49.3
+0.049
Bone_metastasis = 2
−0.0383
ALP = 111
+0.0285
15 other features
+0.028
Total_bilirubin = 11.8
+0.0155
Platelet_count = 158
+0.013
Monocyte_count = 0.5
+0.0128
Lymphocyte_count = 2.25
+0.0117
Eosinophil_count = 0.05
E[f(x)]=0.829
0.75
0.80
0.85
0.90
0.95
Prediction
E[f(x)] = base value | f(x) = model output
Bar length: feature contribution | Direction: sign of effect
